# Outcome of lung metastases due to bone giant cell tumor initially managed with observation

**DOI:** 10.1186/s13018-020-02038-1

**Published:** 2020-11-07

**Authors:** Shinji Tsukamoto, Giovanni Ciani, Andreas F. Mavrogenis, Cristina Ferrari, Manabu Akahane, Yasuhito Tanaka, Michele Rocca, Alessandra Longhi, Costantino Errani

**Affiliations:** 1 Department of Orthopaedic Surgery, Nara Medical University, 840, Shijo-cho, Kashihara-city, Nara, 634-8521 Japan; 2 Orthopaedic Service, IRCCS Istituto Ortopedico Rizzoli, Via Pupilli 1, 40136 Bologna, Italy; 3 First Department of Orthopedics, School of Medicine, National and Kapodistrian University of Athens, 41 Ventouri Street, 15562 Holargos, Athens, Greece; 4 Department of Health and Welfare Services, National Institute of Public Health, 2-3-6 Minami, Wako-shi, Saitama, 351-0197 Japan; 5 Thoracic Surgery Service, IRCCS Istituto Ortopedico Rizzoli, Via Pupilli 1, 40136 Bologna, Italy; 6 Oncology Service, IRCCS Istituto Ortopedico Rizzoli, Via Pupilli 1, 40136 Bologna, Italy

**Keywords:** Giant cell tumor of bone, Observation, Metastasectomy, Denosumab, Metastasis, Lungs

## Abstract

**Background:**

The outcomes of patients with lung metastases from giant cell tumor of bone (GCTB) vary from spontaneous regression to uncontrolled growth. To investigate whether observation is an appropriate first-line management approach for patients with lung metastases from GCTB, we evaluated the outcomes of patients who were initially managed by observation.

**Methods:**

We retrospectively reviewed the data of 22 patients with lung metastases from histologically confirmed GCTB who received observation as a first-line treatment approach. The median follow-up period was 116 months.

**Results:**

Disease progression occurred in 12 patients (54.5%). The median interval between the discovery of lung metastases and progression was 8 months. Eight patients underwent metastasectomy following initial observation. The median interval between the discovery of lung metastases and treatment by metastasectomy was 13.5 months. None of the patients experienced spontaneous regression. Of the 22 patients, 36.4% needed a metastasectomy, and 9.1% required denosumab treatment during the course of the follow-up period. Disease progression occurred in 45.5% of the 11 patients with lung nodules ≤ 5 mm, while all five of the patients with lung nodules > 5 mm experienced disease progression. Progression-free survival was significantly worse in the group with lung nodules > 5 mm compared to the group with lung nodules ≤ 5 mm (*p* = 0.022).

**Conclusions:**

Observation is a safe first-line method of managing patients with lung metastases from GCTB. According to radiological imaging, approximately half of the patients progressed, and approximately half required a metastasectomy or denosumab treatment. However, patients with lung nodules > 5 mm should receive careful observation because of the high rate of disease progression in this group.

## Background

Giant cell tumor of bone (GCTB) accounts for approximately 5% of all primary bone tumors [1]. It usually involves the metaphyseal-epiphyseal region of long bones [2], and its incidence peaks in the third and fourth decade [3]. The main treatment modality is surgery, consisting of curettage or en bloc resection.

Although benign, GCTB is known to be locally aggressive, with a tendency for local recurrence with occasional metastatic potential [4]. GCTBs metastasize in up to 4% of cases, mainly to the lungs [5–16]. While treatment recommendations for lung metastases vary, the most common is a metastasectomy [8, 17–22], though chemotherapy (cytotoxic agents [21, 23], denosumab [24–26], interferon-α [27] or bisphosphonates [28]), radiation [17, 29, 30], and observation [20] have been reportedly used with varying levels of success. For some patients with lung metastases, uncontrolled growth resulting in death has been reported [31]. However, spontaneous regression or growth arrest has also been reported [31], and these patients would benefit from a wait-and-see policy to potentially avoid unnecessary surgical or medical treatments. Before 2010, many authors recommended an immediate metastasectomy when feasible [11, 16, 32–35]. Recently, some authors have proposed that metastasectomy should only be recommended when accompanied by disease progression or symptom development [12, 36–38]. The aim of the wait-and-see policy is to observe the biological behavior of the lung metastases to determine further treatment needs based on disease progression. However, there is limited information regarding the ideal treatment approach for patients with metastatic GCTB.

Therefore, this study aimed to evaluate the clinical outcomes of patients with lung metastases from GCTB initially managed by observation only.

## Methods

**Fig. 1:**
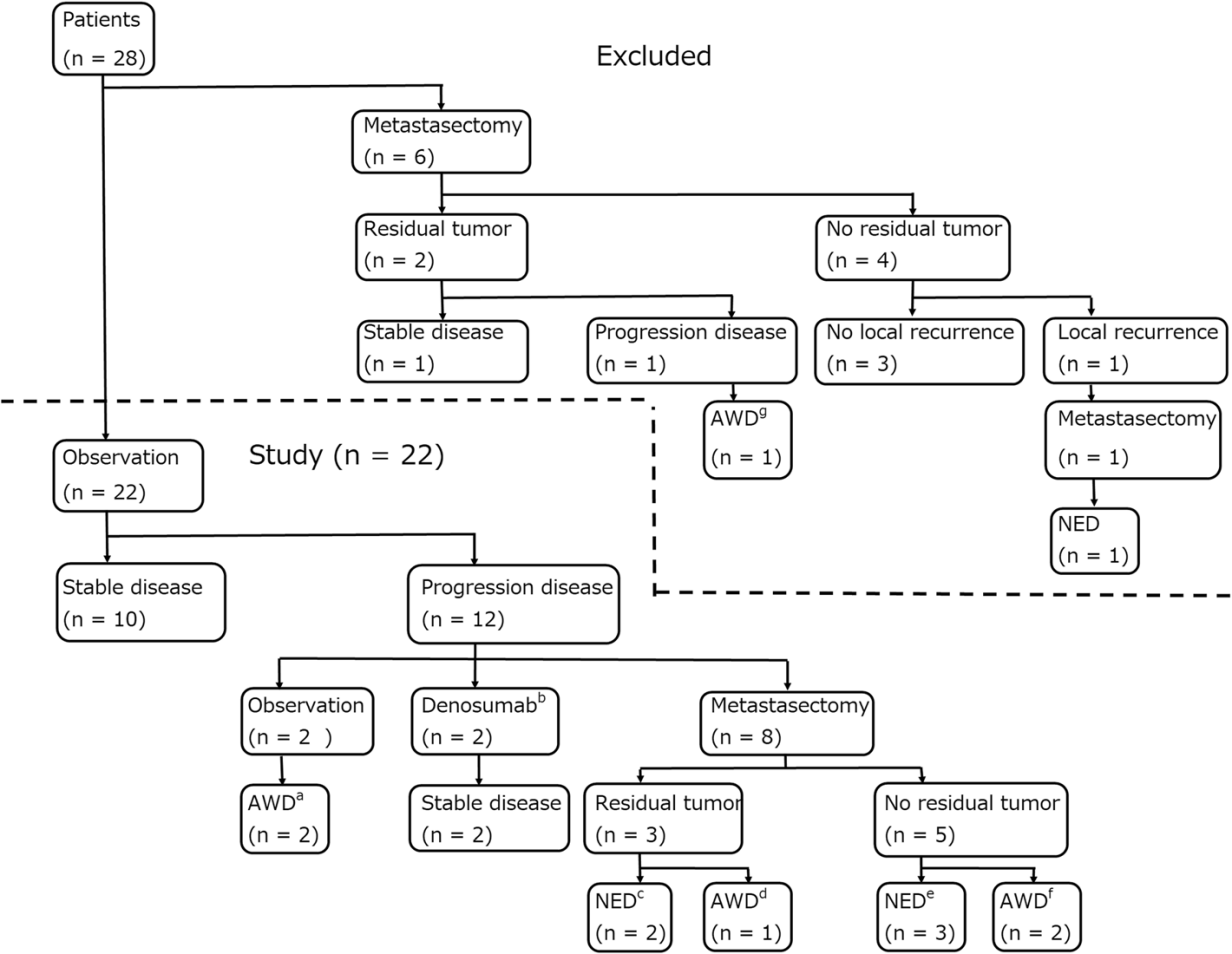
Diagram of patient outcomes. **a** Progression ceased in two patients (case 6, 17). **b** One of these two patients received chemotherapy before starting denosumab treatment and lung lesions progressed under chemotherapy (case 12). **c** One of these patients received a second metastasectomy due to slow progression and never experienced recurrence (case 14), and the other patient received a metastasectomy three more times due to slow progression and never experienced recurrence (case 15). **d** This patient had a lung lesion with slow progression (case 9). **e** One of these 3 patients experienced recurrence and received stereotactic radiotherapy and the lung lesion regressed (case 5), while another patient experienced recurrence and underwent metastasectomy, and no further recurrence was observed (case 10). **f** These two patients experienced recurrences (case 3, 11). **g** Progression ceased in this patient. NED, no evidence of disease; AWD, alive with disease

We retrospectively reviewed the medical records of 496 patients diagnosed with histologically confirmed GCTB in a single institution between 1984 and 2019. Of these 496 patients, 32 developed lung metastases, confirmed by chest computed tomography (CT). Three of these 32 patients were excluded due to malignant transformation of the GCTB. One of the 32 patients was excluded because no information was available regarding the lung nodules. Fourteen patients underwent metastasectomy and were histologically diagnosed with lung metastases from benign GCTB. The other 14 of the 32 patients were diagnosed with lung metastases from benign GCTB after a chest CT showed well-defined and rounded nodular opacities [11, 38]. Six of the 32 patients were excluded because they received a metastasectomy as the first-line approach (Fig. 1). The remaining 22 patients, who had observation as the first-line approach, were included in this study for further analysis (Fig. 1). In the first-line metastasectomy group, five patients received a metastasectomy 1 month after the discovery of lung metastases, and one patient underwent metastasectomy 3 months after the discovery of the lung metastases. Observation as the first-line approach was defined as observation for 4 months or more after the discovery of lung metastases. Metastasectomy as the first-line approach was performed until around 2003, after which, first-line observation was the standard. We evaluated the primary tumor characteristics, as shown in Table 1. Tumors were graded radiographically according to the Campanacci classification system for GCTB [9]. Primary tumor surgeries were performed by curettage or resection. The tumor cavity after curettage was left alone or packed with bone allografts, cement, or cement with bone allografts, as reported in previous studies [39–41]. Resection was indicated for large tumors with soft tissue extension, pathological fractures with joint invasion or an unstable fracture pattern, multiple recurrences, or involvement of expendable bones (head of the fibula or distal end of the ulna), as previously reported [39].

**Table 1:** Details of 22 patients initially managed with observation

Case	Age	Sex	Location of tumor	Campanacci classification	Previous surgery	Lung metastasis at presentation	Pathological fracture at presentation	Surgery for primary tumor	Pre- and postoperative denosumab treatment	Local recurrence
1	24	F	Vertebra	Stage III	No	No	Yes	Curettage	No	No
2	32	F	Distal femur	Stage III	No	Yes	Yes	Resection	No	No
3	26	F	Distal radius	Stage III	No	Yes	No	Resection	No	Yes
4	47	M	Distal femur	Stage III	No	No	No	Resection	No	No
5	28	F	Ilium	Stage II	No	No	No	Resection	Yes	Yes
6	20	F	Distal radius	Stage III	No	No	Yes	Resection	No	No
7	54	F	Phalanx	Stage II	No	No	No	Resection	No	Yes
8	51	F	Distal radius	Stage III	Yes	Yes	Yes	Resection	No	Yes
9	28	F	Proximal tibia	Stage III	No	No	No	Resection	Yes	No
10	27	F	Proximal tibia	Stage III	Yes	No	No	Resection	No	No
11	43	F	Ischium	Stage III	No	No	Yes	Resection	No	Yes
12	17	F	Proximal tibia	Stage III	No	Yes	No	Resection	No	No
13	33	M	Proximal tibia	Stage III	Yes	No	Yes	Resection	No	No
14	38	F	Distal femur	Stage II	Yes	No	No	Resection	No	No
15	28	F	Metacarpal	Stage III	No	No	No	Resection	No	Yes
16	32	M	Metacarpal	Stage III	No	No	No	Resection	No	No
17	37	M	Proximal tibia	Stage III	No	No	No	Curettage	No	No
18	24	F	Proximal fibula	Stage III	No	No	No	Resection	No	Yes
19	25	M	Proximal tibia	Stage II	No	No	No	Curettage	No	Yes
20	36	M	Distal radius	Stage III	No	No	No	Curettage	Yes	Yes
21	28	F	Distal humerus	Stage III	Yes	Yes	No	Curettage	No	No
22	33	F	Distal radius	Stage III	Yes	Yes	No	Curettage	Yes	Yes

After primary tumor treatment, the patients were followed up every 4 months for the first 2 years, every 6 months for the next 3 years, and then annually. Follow-up evaluations included radiographs and a chest CT of the primary tumor area. In cases of lung metastases, lung nodules were observed every 2 months by chest CT. Characteristics of the lung metastases, including the number of lung nodules, the size of the maximum nodule, solitary or multiple lesions, and laterality (unilateral or bilateral) were evaluated, as shown in Table 2. Six of the 22 patients had lung metastases at presentation, and the remaining patients had metachronous metastases. The median interval between surgery for the primary tumor and discovery of lung metastases was 22.5 months (interquartile range [IQR], 0 to 40.8 months). Lung metastasectomy was only indicated for patients with metastatic progression, and all lung metastases were determined to be resectable, with adequate surgical margins and future respiratory function, based on mutual consent among members in a multidisciplinary team conference. The basic procedure for lung resection was wedge resection or segmental resection. Regardless of the number of tumors, their size, or their distribution, surgical resection was indicated for patients who met the abovementioned criteria. In two of the 12 patients with disease progression, the progression ceased while they were awaiting metastasectomy (Fig. 1). Recently, denosumab treatment has been indicated for patients with progression of lung metastases. Eight of the 22 patients underwent metastasectomy and two of the 22 patients received denosumab treatment (120 mg) for 1 year and 11 years, respectively, due to progression of lung metastases (Fig. 1). One of the two patients received chemotherapy (ifosfamide, Adriamycin, interferon, or cyclophosphamide) before starting denosumab treatment (case 12).

**Table 2:** Details of 22 patients initially managed with observation

Case	Interval between surgery of primary tumor and discovery of lung metastasis (months)	Number of lung nodules	Size of maximum nodule (mm)	Lung lesion	Laterality	Progression of lung lesions	Time to progression from discovery of lung metastasis (months)	Treatment for lung lesions	Interval between lung metastasis and metastasectomy (months)	Follow-up period from surgery for primary tumor (months)	Follow-up period from discovery of lung metastasis (months)	Status
1	44	NA	NA	Multiple	Bilateral	No		Observation		139	93	AWD
2	0	4	NA	Solitary	Unilateral	No		Observation		34	33	AWD
3	0	NA	NA	Multiple	Bilateral	Yes	10	Metastasectomy	14	42	42	AWD
4	43	NA	5	Multiple	Bilateral	No		Observation		173	117	AWD
5	16	7	NA	Multiple	Bilateral	Yes	4	Metastasectomy	4	92	75	NED
6	35	NA	10	Multiple	Bilateral	Yes	14	Observation		89	52	AWD
7	22	10	5	Multiple	Bilateral	No		Observation		42	20	AWD
8	0	4	5	Multiple	Bilateral	No		Observation		134	133	AWD
9	23	3	3	Multiple	Bilateral	Yes	11	Metastasectomy	9	52	29	AWD
10	24	5	5	Multiple	Bilateral	Yes	5	Metastasectomy	5	181	155	NED
11	11	6	5	Multiple	Bilateral	Yes	15	Metastasectomy	15	164	150	AWD
12	0	10	10	Multiple	Bilateral	Yes	5	Denosumab		118	116	AWD
13	64	3	NA	Multiple	Bilateral	No		Observation		114	48	AWD
14	11	3	30	Multiple	Unilateral	Yes	6	Metastasectomy	62	141	128	NED
15	30	7	3	Multiple	Bilateral	Yes	66	Metastasectomy	69	118	86	NED
16	23	1	5	Multiple	Bilateral	No		Observation		76	51	AWD
17	46	4	5	Multiple	Bilateral	Yes	3	Observation		120	72	AWD
18	44	NA	NA	Multiple	Bilateral	No		Observation		73	28	AWD
19	10	3	3	Multiple	Bilateral	No		Observation		150	138	AWD
20	40	1	5	Solitary	Unilateral	No		Observation		89	48	AWD
21	0	2	12	Multiple	Bilateral	Yes	5	Metastasectomy	6	183	181	NED
22	0	NA	6	Multiple	Bilateral	Yes	36	Denosumab		87	91	AWD

*NA* not available, *NED* no evidence of disease, *AWD* alive with disease

The size of the lung lesions were assessed by CT and categorized into complete response, partial response, stable disease, or progressive disease, according to the modified Response Evaluation Criteria in Solid Tumors (RECIST) version 1.1, which assesses tumor extent through the sum of the longest diameter of the lesions [42]. At least a 20% increase in the sum of the diameters of the lung nodules or the appearance of one or more new lesions was defined as progressive disease [42]. Progression-free survival was defined as the time from the date of occurrence of lung metastases to the date of lung lesion progression or the last follow-up. Progression-free survival was evaluated with the Kaplan-Meier survival analysis; survival curves were compared with a log-rank test. Significance was defined as *p* < 0.05. Analyses were performed with JMP® 14 (SAS Institute Inc., Cary, NC, USA).

## Results

**Fig. 2:**
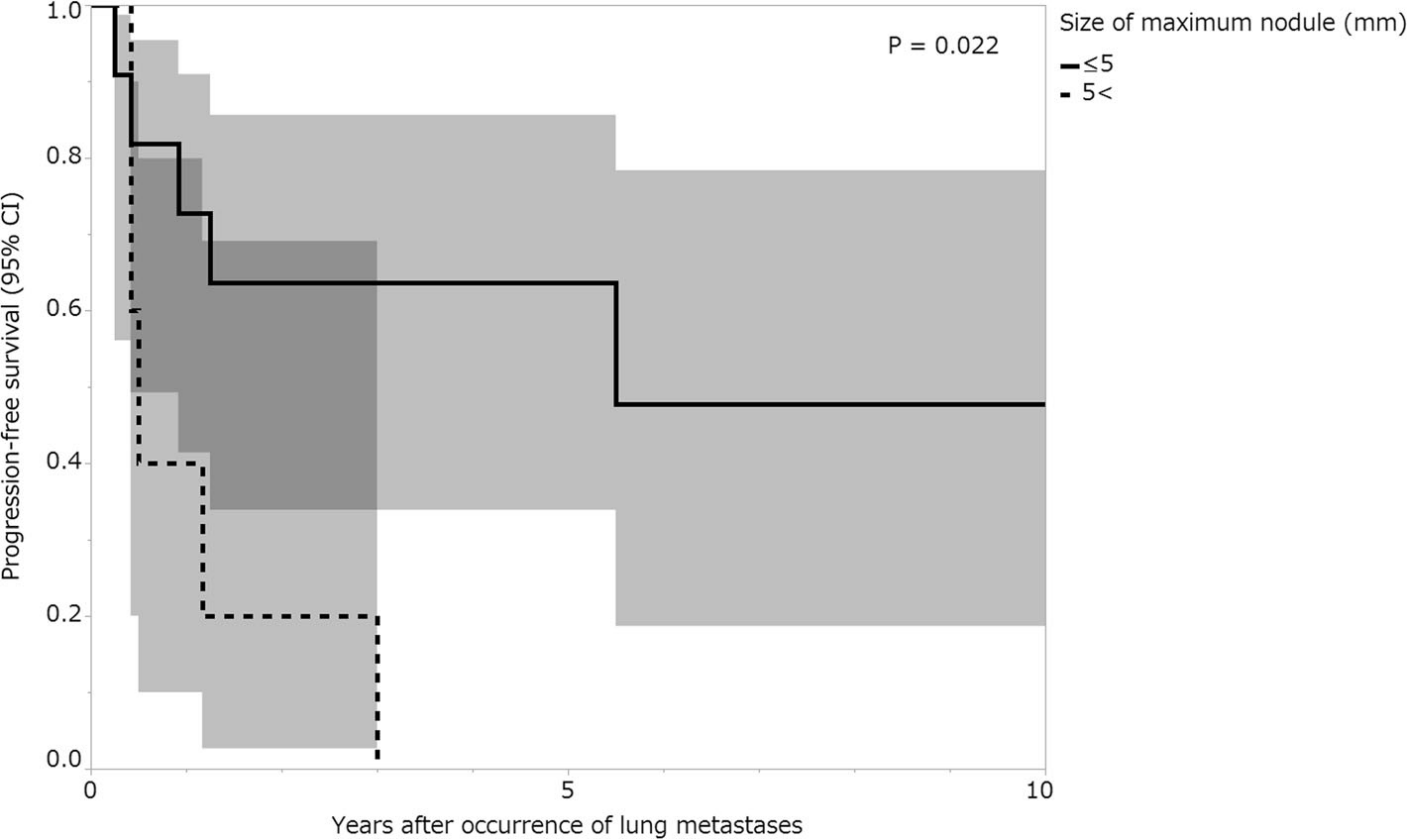
Progression-free survival rates of patients by size of maximum nodule. The shading surrounding the curves shows the 95% confidence intervals (CI)

Patient outcomes are shown in Fig. 1 and Table 2. Disease progression occurred in 12 patients (54.5%), and the median interval between the discovery of lung metastases and progression was 8 months (IQR, 5–14.8 months). In the eight patients who underwent metastasectomy following initial observation, the median interval between the discovery of lung metastases and treatment by metastasectomy was 13.5 months (IQR, 2.8–23.8 months). The median follow-up period after the primary tumor surgery was 116 months (IQR, 74.9–142.9 months). The median follow-up period after the discovery of lung metastases was 80.5 months (IQR, 46.5–129.3 months). With respect to the oncological results, five had no evidence of disease and 17 were alive with lung metastases. None of the patients had died from the disease. Among the 12 patients who did not receive any treatment in the overall therapeutic process, no spontaneous regression occurred. Eight (36.4%) of the 22 patients needed a metastasectomy, and two (9.1%) required denosumab treatment over the course of the follow-up period. Of the 22 patients, 14 did not undergo metastasectomy (63.6%), five patients underwent metastasectomy once (22.7%), and three patients had several metastasectomies (13.6%). Disease progression occurred in five of the 11 patients (45.5%) with lung nodules ≤ 5 mm at the median of 11 months (IQR, 5–15 months) after the discovery of lung metastases, while it occurred in all five patients (100%) with lung nodules > 5 mm at the median of 6 months (IQR, 5–14 months). Progression-free survival was significantly worse in the group with lung nodules > 5 mm compared to the group with lung nodules ≤ 5 mm (*p* = 0.022) (Table 3, Fig. 2). There was no significant difference among any other variables (Table 3). None of the patients with or without disease progression experienced a pleural effusion or respiratory symptoms. None of the patients experienced metastases anywhere other than the lungs. All patients who underwent a metastasectomy had histological confirmation of GCTB lung metastases.

**Table 3:** Univariate analysis for progression-free survival

Variable	No. of patients	5-year progression-free survival (95% CI) (%)	*p* value
Age (years)			
< 30	11	36.4 (14.3–66.1)	0.116
30 ≤	11	60.6 (29.7–84.8)	
Sex			
Male	6	88.3 (36.9–97.7)	0.066
Female	16	32.8 (13.0–61.4)	
Site			
Distal radius	5	40.0 (10.0–80.0)	0.926
Other sites	17	52.9 (30.3–74.5)	
Campanacci classification			
Stage II	4	50.0 (12.3–87.7)	0.913
Stage III	18	48.6 (26.7–71.0)	
Previous surgery			
No	15	60.0 (34.8–80.8)	0.260
Yes	7	28.6 (7.2–67.3)	
Lung metastasis at presentation			
No	16	56.3 (32.4–77.5)	0.502
Yes	6	25.0 (3.8–73.8)	
Pathological fracture at presentation			
No	16	41.7 (20.2–66.9)	0.187
Yes	6	66.7 (26.8–91.6)	
Surgery for primary tumor			
Curettage	6	50.0 (16.8–83.2)	0.769
Resection	16	50.0 (27.3–72.7)	
Pre- and postoperative denosumab treatment			
No	18	55.6 (33.0–76.0)	0.376
Yes	4	25.0 (3.4–76.2)	
Local recurrence			
None	12	41.7 (18.5–69.2)	0.462
≥ 1	10	56.0 (24.7–83.2)	
Interval between surgery of primary tumor and occurrence of lung metastasis (months)			
< 24	13	34.6 (13.2–64.8)	0.399
24 ≤	9	66.7 (33.3–88.9)	
Number of nodules			
< 4	7	57.1 (23.0–85.6)	0.397
4 ≤	9	44.4 (17.7–74.9)	
Lung lesion			
Solitary	2	100.0	0.224
Multiple	20	43.8 (23.9–65.8)	
Laterality			
Unilateral	3	66.7 (15.3–95.7)	0.617
Bilateral	19	46.1 (25.2–68.3)	
Size of maximum nodule (mm)			
≤ 5	11	63.6 (33.9–85.7)	0.022*
5 <	5	40.0 (10.0–80.0)	

*The difference was significant

**Fig. 3:**
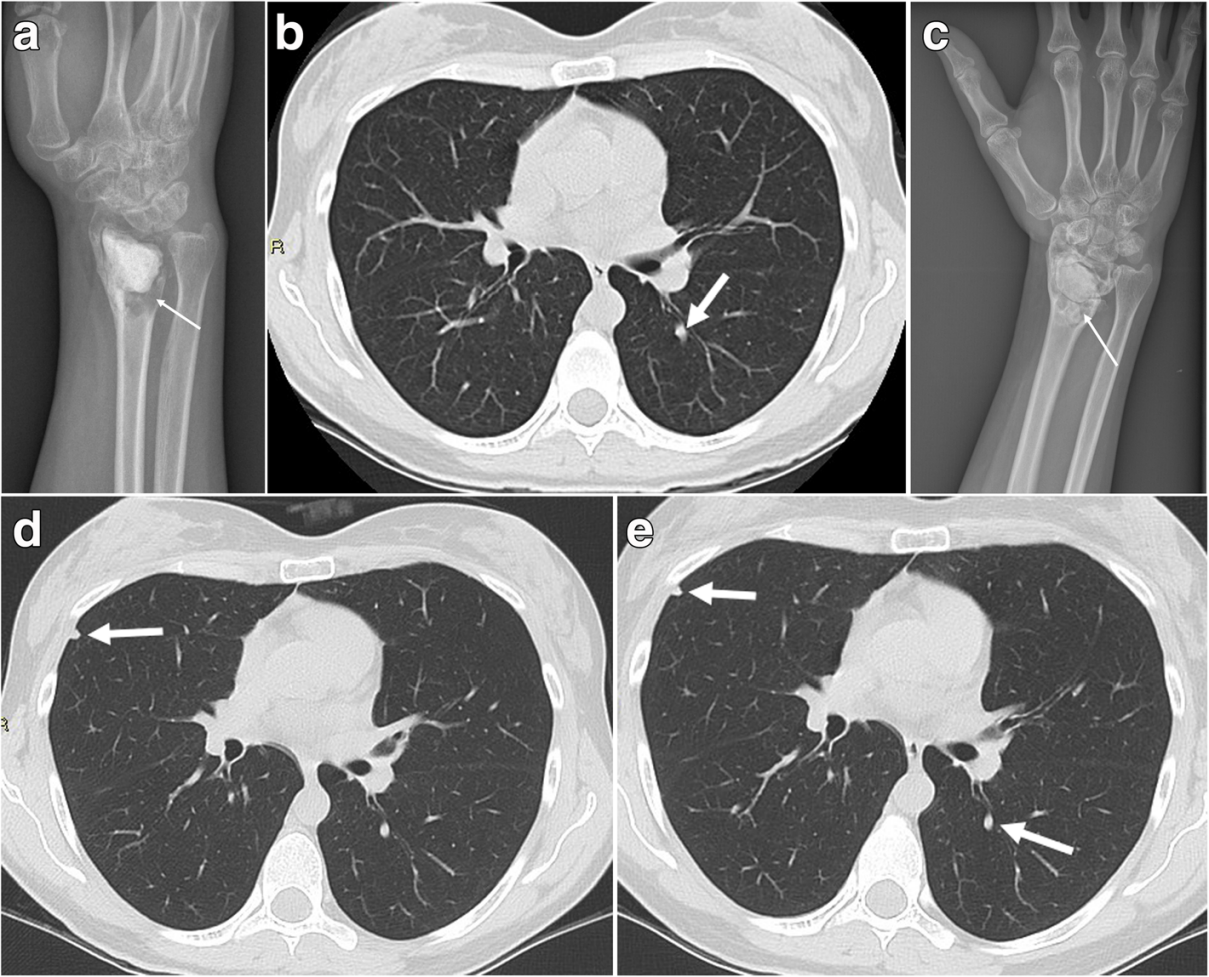
A case of giant cell tumor of bone and lung metastases treated with denosumab rechallenge. She was referred to our institute for local recurrence following curettage and cementing for distal radius giant cell tumor of the bone. Radiograph showed osteolytic lesions (arrow) surrounding the cement (**a**). Chest CT showed lung metastasis in the left lung (arrow) at presentation (**b**). She received preoperative denosumab therapy for 4 months and radiograph showed sclerotic formation (arrow) surrounding the cement (**c**). She then received curettage and cementing and postoperative denosumab therapy for 1 month. However, she experienced local recurrence 14 months after the operation and underwent an en bloc resection and reconstruction with an allograft. Follow-up chest CT showed a new lesion on the right lung (arrow) 3 years after the initial operation at our institute (**d**). She was treated with denosumab for 1 year and the lung lesions were stable (arrows) thereafter (**e**)

Two patients received denosumab treatment due to disease progression. For one of the two patients, chest CT showed disease progression 1 year after the discovery of lung metastases, and the patient was treated with chemotherapy (ifosfamide, Adriamycin, and interferon). Four months after chemotherapy was initiated, chest CT showed continued disease progression, so the patient underwent cyclophosphamide treatment; however, the lung lesions progressed. Denosumab was initiated 4 years after the discovery of lung metastases and continued for 11 years, and the disease remained stable (case 12). In the other patient, chest CT showed lung lesion progression 3 years after curettage for the primary tumor, and the patient was treated with denosumab for 1 year and was observed to have stable disease until the last follow-up. This was a rechallenge because the patient received denosumab treatment before and after curettage of the primary tumor. The patient had no side effects related to denosumab treatment (case 22, Fig. 3).

## Discussion

This study retrospectively analyzed the clinical outcomes of patients with lung metastases resulting from GCTB who were initially managed by observation alone. It was found that 55% of patients experienced disease progression, 45% had stable disease, and none experienced spontaneous regression. Eventually, almost half of the patients needed a metastasectomy or denosumab treatment after the initial observation period (45%). The disease progression rate was higher in the group with lung nodules > 5 mm compared to the group with lung nodules ≤ 5 mm. To the best of our knowledge, this is the first retrospective study to describe the clinical outcomes of lung metastases from GCTB after an initial period of observation. Before 2010, many authors stressed the importance of early detection of metastasis in GCTB with regular and long-term follow-up and, where possible, appropriate immediate surgical resection, such as metastasectomy, wedge resection, or lobectomy was recommended to prevent progressive pulmonary dysfunction [11, 16, 32–35]. On the other hand, a few authors have suggested that pulmonary metastases have a good long-term prognosis and should be kept under observation and aggressive treatments such as lobectomy, chemotherapy, and radiotherapy should be avoided [20, 43]. Since 2015, the authors have suggested that it is unnecessary to perform a lung metastasectomy immediately after the diagnosis of metastasis and that it is more appropriate only when there is disease progression in terms of volume and number of metastases [12, 36–38]. According to a recent systematic review of metastatic GCTB prognoses, spontaneous regression was observed in 4.5% of patients [44]. There is often no change in volume with GCTB lung metastases [36]. Since it is impossible to predict the behavior of these metastases [38], it is reasonable to evaluate the tumor biology with observation in each case in order to decide on further treatments, such as a metastasectomy or medical treatments. However, patients with lung nodules > 5 mm require careful observation due to the high rate of disease progression.

The mortality of patients with metastases from GCTB who underwent metastasectomy ranged from 0 to 50% [2, 12–14, 20, 31, 32, 34, 37, 45–50]. The recurrence rate of patients with metastases from GCTB who underwent metastasectomy ranged from 0 to 50% [2, 13, 14, 20, 31, 32, 37, 45, 46, 49]. The outcomes following metastasectomy varied due to the unpredictable tumor behavior of the GCTB lung metastases. Studies have shown that an aggressive lung metastasectomy might fail to result in a cure [51].

Since reporting on the efficacy of cytotoxic chemotherapy for lung metastases from GCTB is limited [34, 52], its role is not well defined. However, considering that GCTBs are borderline tumors, they are not responsive to chemotherapy even after the appearance of the lung metastases [48]. There is anecdotal evidence that interferon α-2a can be effective in stabilizing progressive GCTB refractory to other modalities such as surgery, radiation, and cytotoxic chemotherapy [53–55]. Interferon may have activity in GCTBs via its antiangiogenic properties. Interferon, however, is not well tolerated and is associated with numerous side effects, including depression and ischemic events [56].

Feigenberg et al. [57] reported three patients with lung metastases from GCTB who were treated with whole-lung radiotherapy. One patient’s lung metastasis progressed after treatment, and the patient soon died. The other two patients were long-term survivors (7.5 years and 13 years) with complete resolution of detectable disease. However, radiotherapy may induce secondary malignant transformation, which is a concern, especially because most patients are relatively young. The reported risk of malignant transformation varies between 0 and 5% [58–62].

Denosumab was capable of stopping the progression of lung metastases in two patients. For one of these patients, lung metastases progressed despite chemotherapy, but denosumab halted the progression of lung metastases. Palmerini et al. [26] reported a series of 15 patients with metastatic GCTB treated with denosumab, and all achieved tumor control. Engellau et al. [63] reported on 38 patients with metastatic GCTB who achieved tumor control with denosumab treatment. Thus, denosumab could halt disease progression in most metastatic GCTBs. In our study, two patients underwent denosumab treatment and achieved tumor control of lung metastases without side effects. In one of these 2 patients, denosumab was administered twice before surgical management of the primary lesion and then for the treatment of lung nodules. Each of the two denosumab treatments was effective for the patient. However, to date, only two cases have demonstrated that denosumab rechallenge could be effective [64].

Balke et al. [28] reported a series of 12 patients with metastatic GCTB who had stable disease following bisphosphonate treatment. Bisphosphonate is also a treatment option for lung metastases from GCTB.

Currently, based on the aforementioned studies and the findings of this study, we determined that observation of the biological behavior of lung metastases is a first-line approach. If the lung metastases progress, denosumab should be administered once every 4 to 6 months to reduce the risk of complications such as osteonecrosis of the jaw [26]. If the denosumab must be discontinued due to complications and the lung metastases progress again, a metastasectomy should be performed. If the lung metastases are inoperable or the patient refuses metastasectomy, a denosumab rechallenge after the patient recovers from the complication or stereotactic radiotherapy treatment is recommended.

This study has several limitations. First, we have histological documentation of lung nodules only for patients who underwent resection of their lung metastases. However, most patients with GCTB are healthy and young and infrequently have lung lesions; therefore, these lung lesions, when observed on imaging studies of GCTB patients, most likely represented GCTB lung metastases. Second, because information on the size of lung nodules was not available in six of the 22 patients, the association between the size of the lung nodule and disease progression should be interpreted with caution. Third, a power analysis was not performed, and there was a risk of type II error due to the small sample size. If an adequate number of patients is gathered in the future, significant differences may appear regarding the other variables in this study. Forth, this is a retrospective study, and patients were treated differently over the long-term following multidisciplinary team meetings. The treatments have also changed in relation to the discovery of new therapies such as denosumab. However, based on the results of this study, we can now recommend a therapeutic strategy for the treatment of lung metastases from GCTB.

## Conclusions

This study showed that observation can be used safely as a first-line management approach for patients with lung metastases from GCTB. According to radiological imaging, approximately half of the patients progressed, and approximately half required some form of treatment. However, patients with lung nodules > 5 mm should be carefully observed because of the high rate of disease progression in this group.

## Data Availability

The datasets generated and/or analyzed during the current study are not publicly available due to privacy considerations but are available from the corresponding author upon reasonable request.

## Abbreviations

GCTB: Giant cell tumor of bone
CT: Computed tomography
IQR: Interquartile range
RECIST: Response Evaluation Criteria in Solid Tumors
